# Gut microbiota metabolite acetate mediates free fatty acid receptor 2 expression to alleviate atopic dermatitis

**DOI:** 10.3389/fmicb.2025.1595532

**Published:** 2025-08-12

**Authors:** Siqi Ye, Jingwen Wang, Feng Luo, Jinjing Jia, Xiumei Mo, Dacan Chen

**Affiliations:** ^1^Department of Dermatology, The Second Affiliated Hospital of Guangzhou University of Chinese Medicine, Guangzhou, China; ^2^State Key Laboratory of Dampness Syndrome of Chinese Medicine, The Second Affiliated Hospital of Guangzhou University of Chinese Medicine, Guangzhou, China; ^3^The Second Clinical School of Guangzhou University of Chinese Medicine, Guangzhou, China

**Keywords:** atopic dermatitis, gut microbiota, short-chain fatty acids, acetate, free fatty acid receptor 2

## Abstract

**Introduction:**

Previous studies have demonstrated that gut microbiota and its metabolites, short-chain fatty acids (SCFAs), are involved in the inflammatory manifestations and immune regulation of atopic dermatitis (AD). However, their potential associations and mechanisms remain unclear.

**Methods:**

This study used antibiotics to construct a mouse model to analyze the performance of AD mice after gut microbiota destruction. 16S rRNA amplicon sequencing combined with HM700 high-throughput metabolomics was used to characterize differential microbial components and key metabolites in fecal specimens of AD murine models. Moreover, the mechanism of action of the key metabolite was investigated.

**Results:**

After antibiotic treatment, AD murine models demonstrated exacerbated clinical manifestations, characterized by enhanced dermatitis severity, significant ear edema, and elevated inflammatory responses. 16S rRNA sequencing revealed significant changes in the *Bacteroidetes/Firmicutes* ratio, while HM700 identified acetate as an important regulatory metabolite in AD mice. Acetate supplementation in AD mice significantly ameliorated 2,4-dinitrobenzene (DNCB)-induced dermatitis, as evidenced by reduced skin lesion severity, lower dermatitis scores, and decreased epidermal thickening. Mechanistically, acetate attenuated allergic responses by binding to free fatty acid receptor 2 (FFAR2) and suppressing the Th2 pathway through GATA binding factor 3 downregulation, along with marked reductions in serum immunoglobulin E and thymic stromal lymphopoietin levels. Notably, acetate administration did not alter gut microbiota composition or relative abundances.

**Conclusion:**

Our results revealed that the ratio of *Bacteroidetes/Firmicutes* and low levels of acetate play important regulatory roles in AD, and exogenous supplementation of acetate can alleviate DNCB-induced AD in mice through the FFAR2 and Th2 pathways. These findings provide valuable insights into the mechanisms of AD occurrence and progression, microbial community dynamics, metabolic regulation, and functional food innovation.

## Highlights

- *The Bacteroidetes/Firmicutes* ratio plays an important regulatory role in AD
- Gut microbial metabolite acetate plays a vital role in AD pathogenesis
- These effects are regulated through the FFAR2 and Th2 pathway

## 1 Introduction

Atopic dermatitis (AD) is a chronic, recurrent inflammatory skin disorder primarily manifested by persistent inflammation, frequent relapses, skin barrier impairment, and immune dysregulation (Yepes-Nuñez et al., 2023; Laughter et al., 2021; Werfel et al., 2024). Epidemiological data indicate that the prevalence of AD in children ranges from 15% to 30%, while adults exhibit a lower rate at ~10% (Laughter et al., 2021). Recurrent inflammation, long-term course, and persistent scratching can lead to sleep disorders, disrupting daily activities and reducing quality of life. Changes in microbiome composition can increase the risk of atopic diseases, including AD (Zubeldia-Varela et al., 2022). The diversity of the gut microbiota in patients with AD was significantly decreased and negatively correlated with disease severity (Moniaga et al., 2022). Similarly, our previous study demonstrated that changes in microbiota composition correlated with patients with AD, and *Bacteroidaceae* may be a potential biomarkers associated with its diagnosis (Ye et al., 2021). Microbiota-derived treatments, such as fecal microbiota transplantation and probiotics are beneficial for patients with AD (Kim et al., 2021; Fang et al., 2021). Emerging as a novel therapeutic approach for AD, fecal microbiota transplantation demonstrated remarkable potential in modulating the composition, structural organization, and functional diversity of the gut microbiota in patients (Liu et al., 2024). Moreover, it can increase the levels of short-chain fatty acids (SCFAs) and metabolic products in the gut of AD mice, restore Th1/Th2 balance through gut microbiota, and improve allergic reactions. SCFAs are produced by colonic microbial fermentation of dietary fibers, which are carboxylic acids with < 6 carbon atoms, such as acetate, butyrate, and propionate (Maslowski et al., 2009; Yao et al., 2022). SCFAs are closely associated with AD pathogenesis, and fecal SCFA levels from patients with AD are generally lower than those in healthy individuals (Reddel et al., 2019; Kim et al., 2019). Case-control studies have shown that SCFA levels in breast milk and childhood AD consistently negatively correlated with acetic acid levels after adjusting for confounders (Wang et al., 2022). Butyrate can regulate immune cell homeostasis and alleviate excessive granulocyte drive in inflammatory disease models, while propionate can reduce itching and skin inflammation by regulating sensory transient receptor potential channels and neuropeptide secretion in the dorsal root ganglion (Dang et al., 2023; Xu et al., 2024). SCFAs modulate immune homeostasis, suppress inflammatory pathways, and reinforce intestinal epithelial barrier function through G-protein coupled receptor (GPCR) activation and histone deacetylase inhibition (Xiao et al., 2023; Corrêa-Oliveira et al., 2016; Smith et al., 2013). Increasing evidence suggests that SCFAs play a protective role against allergic diseases, particularly AD, asthma, and food allergy. However, their effect on the host differs by type, and the association between individual SCFAs and specific allergic conditions varies, with the underlying regulatory mechanisms remaining largely unknown. Although changes in the gut microbiota and SCFAs have been identified in patients with AD, the direct or indirect interactions between them remain unclear and warrant further investigation. Recurrent rash attacks can be frustrating to patients with AD. If effective dietary supplements or probiotics can prevent recurrence, its application prospects will be extensive. Previous study demonstrated that dietary fiber intake was crucial for promoting skin barrier function; an intact skin barrier can prevent allergen penetration and mitigate allergic reactions (Trompette et al., 2022). Therefore, in-depth research on the interaction and relationship between gut microbiota and SCFAs, the metabolites derived from dietary fiber fermentation, is important for prevention and treatment of AD. This study aimed to search for taxonomic and functional microbial community profiles along with metabolic signatures in AD mice, as well as investigate the relationship between them and related mechanisms in AD.

## 2 Materials and methods

### 2.1 Main reagents and materials

Ampicillin, neomycin, metronidazole, vancomycin, 2,4-dinitrobenzene (DNCB), and sodium acetate anhydrous were purchased from Sigma Chemical Co. (St. Louis, MO, USA). Mouse enzyme-linked immunosorbent assay (ELISA) kits for immunoglobulin E (IgE) and thymic stromal lymphopoietin (TSLP) were all obtained from Abcam (Cambridge, MA, USA). TRIzol reagent, SYBR Green Master Mix, Microamp Fast Optical 96-Well Reaction Plate, and primary antibody against free fatty acid receptor 3 (FFAR3) as well as peroxidase-conjugated secondary antibodies were acquired from Thermo Fisher Scientific (Wilmington, DE, USA). Primary antibodies against GATA binding factor 3 (Gata3) and β-actin were obtained from Santa Cruz Biotechnology (Santa Cruz, CA, USA). Primary antibody against free fatty acid receptor 2 (FFAR2) was obtained from Alomone Labs (Alomone Labs, Jerusalem, Israel).

### 2.2 Experimental animals

The animals used were six-to-eight-week-old male BALB/c mice (20 ± 2 g), which were obtained from the Guangdong Medical Laboratory Animal Center (China) and subjected to a 7-day acclimation period in a controlled animal facility maintained at 22 ± 3°C, with 55 ± 5% humidity, and a 12-h light/dark cycle.

### 2.3 Establishment of the AD and gut microbiota depletion mouse model

**Figure 1:**
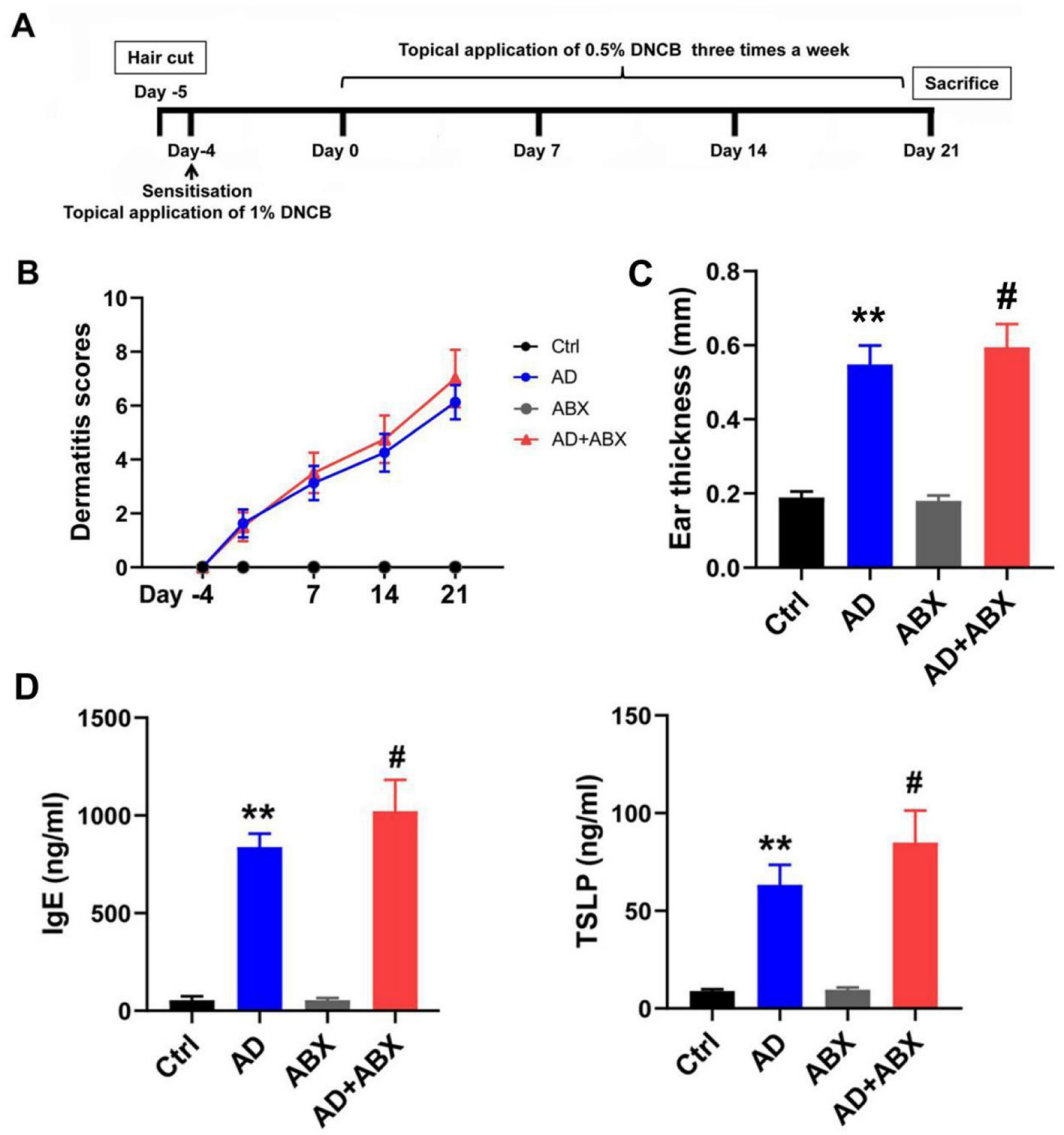
ABX treatment can exacerbate symptoms of AD mice. **(A)** Experimental scheme for the DNCB-induced AD mice. **(B)** Dermatitis scores of the mice. **(C)** Mouse ear thickness among Ctrl, AD, ABX, and AD+ABX mice. **(D)** Levels of IgE and TSLP in the peripheral blood of mice. The results are presented as mean ± SEM (*n* = 6–8). **p < 0.01 vs. Ctrl; #*p* < 0.01 vs. AD.

AD-like dermatitis was induced in mice by applying DNCB (Sigma-Aldrich) to the dorsal skin and ears (Figure 1A). After complete hair removal via depilation, 200 μL of 1% (w/v) DNCB solution was challenged for sensitization. Beginning on day 0 (4 days post-sensitization), the ears and dorsal skin were applied with 0.5% DNCB solution three times weekly for three consecutive weeks. The control group was treated with a vehicle. Anesthesia was performed by isoflurane induction. On day 21, the mice were sacrificed using pentobarbital sodium. Blood, dorsal skin, spleens, and colonic tissue samples, as well as intestinal contents, were collected. An electronic digital caliper was used to measure and record mouse ear thickness. Dorsal skin, spleens, and colonic tissue samples, as well as intestinal contents, were collected. Dermatitis scores were assessed at days−5, 0, 7, 14, and 21 by scoring skin lesions according to symptoms, including erythema/bleeding, dryness/scaling, edema, and erosion/scratching. The dermatitis severity was scored from 0–3 points, indicating no, mild, moderate, and severe symptoms, respectively. The final score was the sum of the four symptom scores. An investigator blinded to the grouping conducted the assessments.

We established a mouse model with gut microbiota depletion to investigate the role of the gut microbiota in AD. The specific pathogen-free mice were randomly divided into the following four groups: control (Ctrl), AD model (AD), antibiotic exposure control (ABX), and AD+antibiotic exposure (AD+ABX) groups. The ABX and AD+ABX mice were fed drinking water containing ampicillin (1 g/L), neomycin (1 g/L), metronidazole (1 g/L), and vancomycin (0.5 g/L) ad libitum 2 days before the establishment of the AD model, with antibiotic intervention for 28 days (Hong et al., 2019). Subsequently, we used 16S rRNA sequencing and metabolomics to screen for the differential microbiome components and metabolites in the feces of the AD mice.

### 2.4 Sodium acetate treatment

After screening and identifying the key SCFA as acetate, the mice were categorized into the following four groups: control (Ctrl), AD model (AD), sodium acetate supplementation (Ac), and AD+acetate supplementation (AD+Ac) groups. We added sodium acetate (NaOAc) (200 mM, Sigma-Aldrich, catalog number: S2889) to the drinking water from the beginning of the experiments (day−5) to day 21 for Ctrl+Ac and AD+Ac mice, similar to the experiments performed with diet (Antunes et al., 2019).

### 2.5 DNA extraction and library construction

Fresh fecal pellets (~150–200 mg/mouse) were collected and frozen in liquid nitrogen within 1 h after sampling until further analysis. Microbial community DNA were extracted and quantified. The quality of the DNA was verified and the V3-V4 hypervariable region of the bacterial 16S rRNA gene was amplified. Purified PCR amplicons were obtained using Agencourt AMPure XP beads (Beckman Coulter, USA) with an optimized elution buffer, followed by quality control on an Agilent 2100 Bioanalyzer (Agilent Technologies, USA). The DNA samples were sequenced on the DNB MGI-2000 platform (BGI, Shenzhen, China) to generate a 2 × 300 bp paired-end reads.

### 2.6 Microbial analysis

Raw sequencing data underwent quality control to remove low-quality bases, adapter sequences, and reads with >5% N-content, resulting in high-quality clean reads. The paired-end sequencing data were processed through VSEARCH (version 2.13.6) to generate consensus sequences, employing a stringent 97% similarity criterion, followed by clustering analysis to identify operational taxonomic units (OTUs). Representative OTU sequences were classified according to SILVA or Greengenes 16S rRNA reference database. Microbial alpha diversity was quantified using Faith's phylogenetic diversity metric, while LEfSe analysis (LDA effect size ≥3.0) was conducted to identify discriminative features at different taxonomic ranks.

### 2.7 HM700 high-throughput targeted metabolomics

Fresh fecal pellets from Ctrl and AD mice were analyzed using a Waters UPLC I-Class Plus system (Waters, USA) coupled with a QTRAP 6500 Plus triple quadrupole mass spectrometer (SCIEX, USA) (Liang et al., 2024). The Skyline software suite (MacCoss Lab, University of Washington) was used for targeted metabolite quantification in fecal extracts, followed by multivariate statistical analysis. Prior to principal component analysis (PCA), the data were log2-transformation and normalized using Pareto scaling. To evaluate metabolite-class associations, partial least squares (PLS) regression was implemented to assess the discriminatory power of metabolite expression profiles, enabling the evaluation of their impact on sample classification. Metabolites demonstrating significant differential abundance were determined based on a two-fold threshold criterion (≥1.2-fold increase or ≤ 0.83-fold decrease) with statistical significance set at *P* < 0.05.

### 2.8 SCFA determinations

Quantification of SCFA-containing ether fractions was performed using liquid chromatography-mass spectrometry (LC-MS). Following extraction, sample analysis was conducted on a Waters UPLC I-Class Plus system (Waters Technologies, MA, USA) integrated with a QTRAP 6500 Plus mass spectrometer. Concentration determination was achieved through an external standard calibration protocol covering the relevant concentration range.

### 2.9 Chromatography conditions and mass spectrometry conditions

The chromatographic separation was conducted using a Waters ACQUITY Premier BEH C18 column (2.1 × 50 mm, 1.7 μm) with a gradient elution program configured as detailed below: mobile phase B (containing 0.1% formic acid) was maintained at 15% for 1.0 min, linearly increased to 55% over 4.0 min, and then rapidly reversed to the initial concentration at a rate of 0.5%/min within 1.0 min (5.1–6.0 min). The system was operated under the following optimized parameters: column temperature set to 40°C (midpoint of the manufacturer's recommended range, 20–90°C), and flow rate maintained at 0.35 mL/min.

The QTRAP 6500 Plus mass spectrometer, equipped with a Turbo Ion Spray interface, was operated in negative ion mode with the following source parameters: ion source temperature of 550°C, ion spray voltage at −4,500 V, auxiliary gases (gas I and II) at 50 psi, and curtain gas at 30 psi. Data acquisition was performed using multiple reaction monitoring (MRM), with collision energy and declustering potential optimized for each analyte based on their physicochemical properties. MultiQuant (SCIEX, USA) was used to identify and quantify the metabolites. The resulting data matrix for obtaining information includes metabolite identification and quantitative results was processed for analysis.

### 2.10 ELISA analysis

IgE and TSLP were determined using ELISA kits with the supernatant obtained from mouse blood samples after centrifugation. Quantitative analysis involved duplicate measurements of absorbance at 450 nm using a standardized microplate reader system.

### 2.11 Histopathological analysis

All dorsal skin tissue samples were fixed with 4% paraformaldehyde, underwent routine dehydration, and was subsequently embedded in paraffin. The prepared paraffin sections were stained with hematoxylin and eosin (H&E) and subjected to histopathological observation using an Olympus BX53 light microscope.

### 2.12 Real-time quantitative PCR analysis

RNA was extracted from the colon, and cDNA synthesis was performed. Quantitative PCR was conducted using SYBR Green Master Mix on a ViiA 7 PCR system (Applied Biosystems). Glyceraldehyde 3-phosphate dehydrogenase (GAPDH) was selected as the housekeeping reference gene, with the QPCR data quantified through the following formula: Relative mRNA expression = 2^(Δ *Ct target gene*−Δ*Ct GAPDH*)^. The primer sequences used were: FFAR3: Forward 5′-AGTCGCCTGGTGTGGATACTGAG-3′, Reverse 5′-GCCGAAGCAGACGAAGAAGATGAG-3′; FFAR2: Forward 5′-GCTGACAGGCTTCGGCTTCTAC-3′, Reverse 5′-CAGAGCAGCGATCACTCCATACAG-3′; GPR109a: Forward 5′-TGAGGCAGAGACAGATGGACAGAC-3′, Reverse 5′-GAGAAGCCAGAAGATGCGGATGC-3′; T-bet: Forward 5′-AGCCGTTTCTACCCCGAC-3′, Reverse 5′-GCTCACAGCTCGGAACTCC-3′; Gata3: Forward 5′-TCTGGAGGAGGAACGCTAATGGG-3′, Reverse 5′-CGGGTCTGGATGCCTTCTTTCTTC-3′; RORγt: Forward 5′-TGTCCCGAGATGCTGTCAAGTTTG-3′, Reverse 5′-TCCTGTTGCTGCTGCTGTTGC-3′; Foxp3: Forward 5′-AAGAATGCCATCCGCCACAACC-3′, Reverse 5′-TACGGTCCACACTGCTCCCTTC-3′; GAPDH: Forward 5′-TCCACTCACGGCAAATTCAAC-3′, Reverse 5′-GTAGACTCCACGACATACTCAGC-3′.

### 2.13 Western blot (WB) analysis

Mouse colon tissues were homogenized and processed for protein extraction, with concentrations subsequently quantified following the manufacturer's guidelines. Protein samples were fractionated by 8–12% SDS-PAGE and subsequently transferred onto polyvinylidene difluoride (PVDF) membranes pre-activated with methanol. Following blocking and washing, the membranes were incubated overnight with anti-Gata3, anti-FFAR3, anti-FFAR2, and anti-β-actin primary antibodies. After incubation with the secondary antibody at room temperature and three washes, protein signals were visualized using a Bio-Rad GelDoc imaging system.

### 2.14 Statistical analysis

Statistical analysis was performed using GraphPad Prism 8.0.2 (San Diego, CA, USA). The experimental results are presented as mean ± standard error of the mean for normally distributed data, while quartiles were used for non-normally distributed data. One-way analysis of variance, followed by Tukey's *post-hoc* multiple comparisons, was used. Statistical significance was determined at *P* < 0.05.

## 3 Results

### 3.1 Disruption of gut microbiota can exacerbate symptoms of AD mice

To evaluate whether the gut microbiome mediates the protective effects in AD, we administered oral antibiotics to mice for 28 days. The alpha diversity index reflects the abundance and diversity of microbial communities in samples through five indices, namely observed species, Chao, Ace, Shannon, and Simpson. The observed species, Chao, and Ace indices reflect the species richness index, while the Shannon and Simpson values indicate the species diversity of a community. The complexity of the sample is directly proportional to the first four values and negatively correlated with the Simpson value.

No significant difference was observed in the alpha diversity index between the Ctrl and AD groups, while antibiotic treatment significantly inhibited the gut microbiota of mice, affecting diversity and abundance (Table 1). The antibiotic-treated mice showed dehydration and weight loss in the early stages owing to their resistance to antibiotics in their drinking water. The ABX and AD+ABX mice gradually recovered on the 10th day, and their weights did not differ significantly from the other groups at the final endpoint (data not shown). Skin lesions, ear swelling, and increased serum IgE and TSLP expression are the main clinical symptoms of AD. The Ctrl and ABX groups did not receive DNCB, and their dermatitis scores were zero at all time points. Dermatitis (*p* > 0.05) and ear thickness (*p* < 0.05) were more severe in the AD+ABX mice than in the control group (Figures 1B, C). Hair regrowth in most mice after day 14 hindered accurate assessment of erythema and hemorrhage due to limitations in the dermatitis scoring system. The IgE and TSLP levels increased significantly after ABX treatment with AD mice (Figure 1D). Therefore, reduction in skin inflammation and allergies depends on the preserved intestinal microbiota.

**Table 1:** Alpha diversity comparison results after exercise intervention.

**Bacterial index**	**Sobs (mean ±SD)**	**Chao (mean ±SD)**	**Ace (mean ±SD)**	**Shannon (mean ±SD)**	**Simpson (mean ±SD)**	**Coverage (mean ±SD)**
Ctrl	363.90 ± 25.15	403.90 ± 31.15	397.00 ± 28.57	4.07 ± 0.21	0.04 ± 0.01	1.00 ± 0.00
AD	386.80 ± 27.49	432.71 ± 30.35	424.40 ± 30.93	4.26 ± 0.15	0.04 ± 0.01	1.00 ± 0.00
ABX	58.17 ± 18.80^**^	117.97 ± 27.54^**^	141.35 ± 32.92^**^	1.17 ± 0.56^**^	0.40 ± 0.07^**^	1.00 ± 0.00
AD + ABX	54.86 ± 15.78^**^	122.57 ± 47.33^**^	189.49 ± 98.78^**^	0.92 ± 0.26^**^	0.45 ± 0.06^**^	1.00 ± 0.00

^**^P < 0.01 vs. AD.

### 3.2 *Bacteroidetes/Firmicutes* ratio changed significantly in AD mice

**Figure 2:**
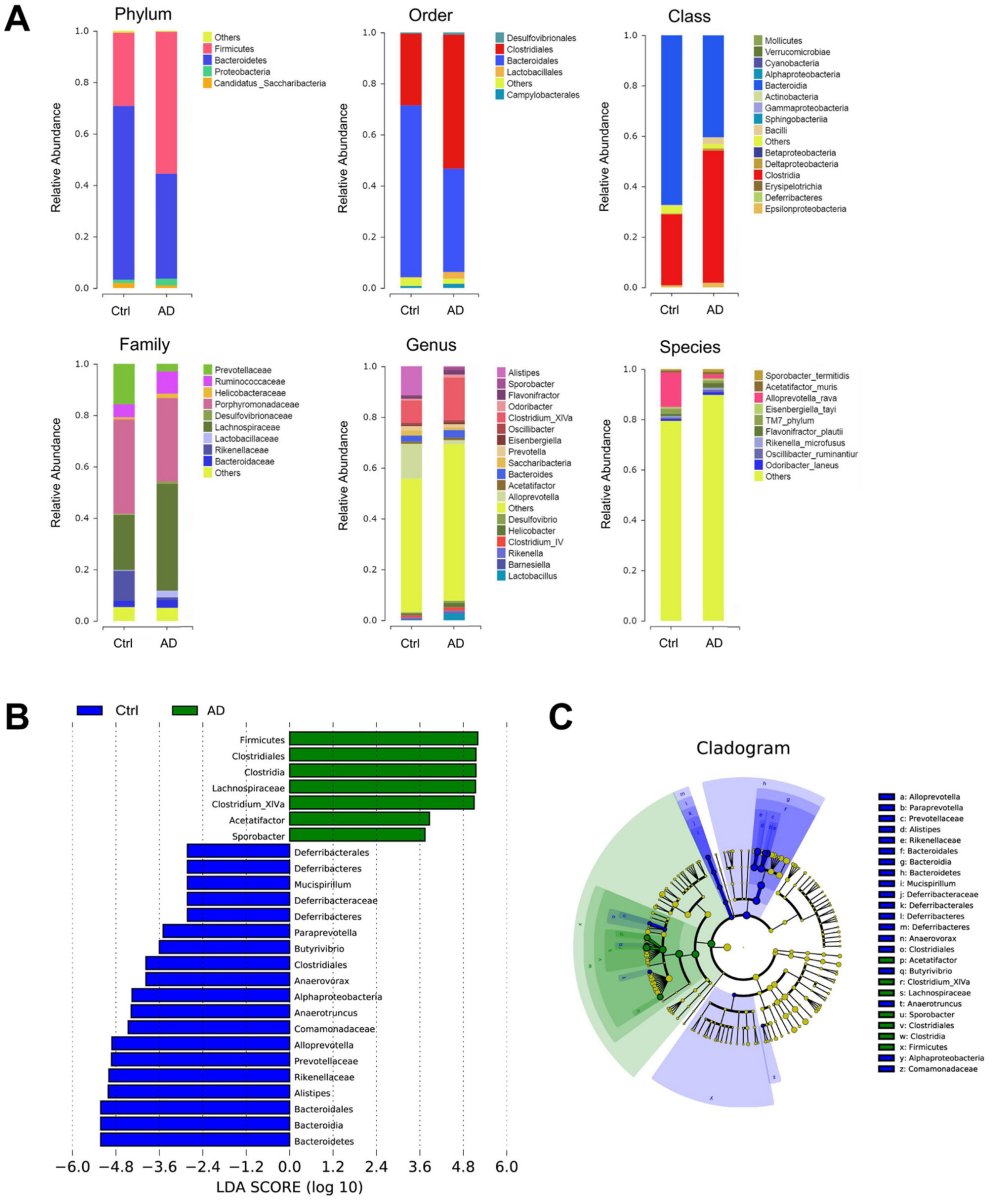
16S sequencing analysis in Ctrl and AD mice. **(A)** Relative abundance of the fecal microbiota composition from Ctrl and AD mice at six levels. **(B)** An LDA was conducted to identify the differentially abundant bacteria between Ctrl and AD mice. **(C)** LEfSe analysis. The results are presented as mean ± SEM (*n* = 8).

**Figure 3:**
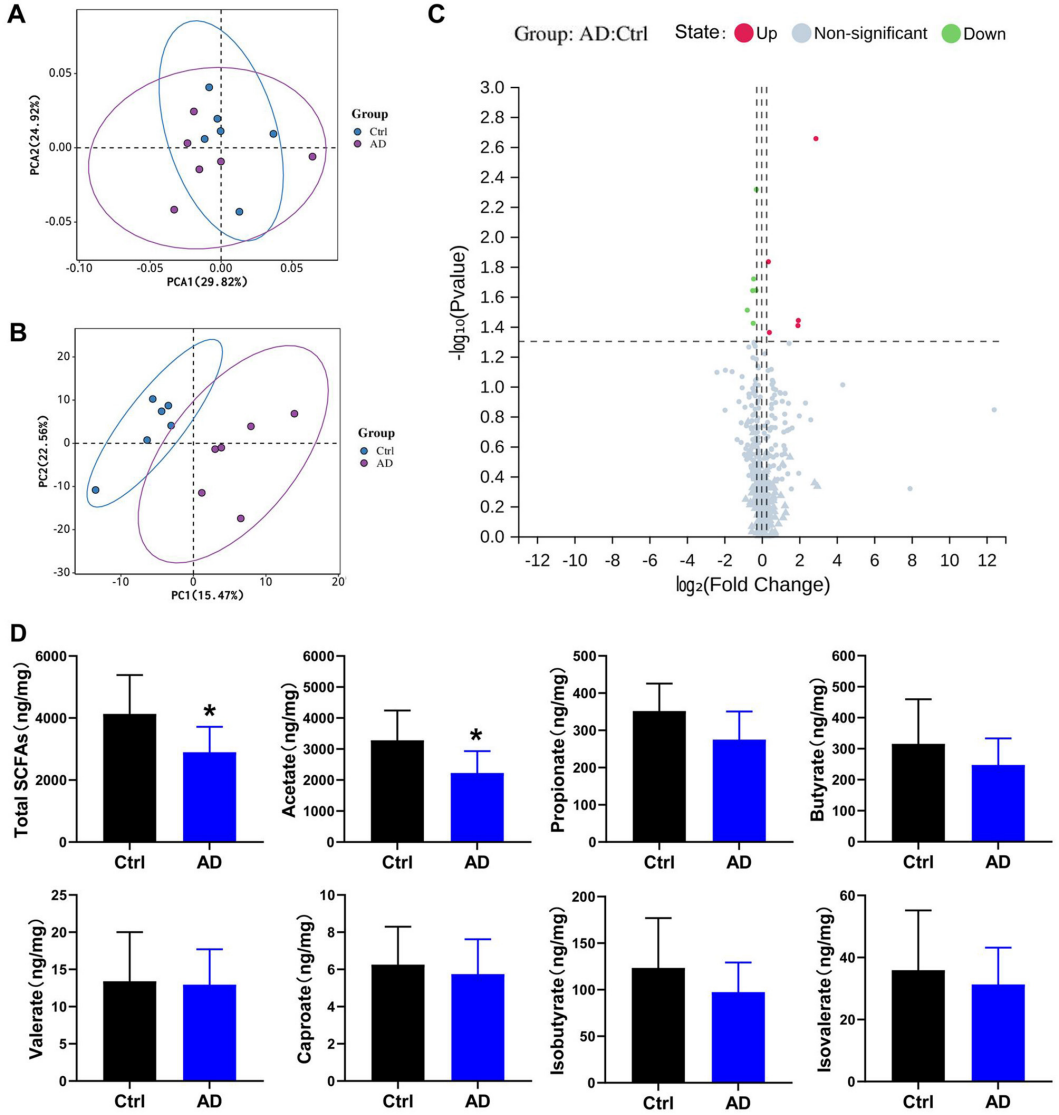
Differential metabolite analysis results in Ctrl and AD mice. **(A)** PCA score plots. **(B)** PLS-DA model in Ctrl and AD mice. **(C)** Volcano plot of metabolites. Red indicates upregulated, blue indicates down-regulated, and gray marks metabolites with no significant changes. **(D)** Quantitative analysis of SCFA in fecal samples (ng/mg feces). The results are presented as mean ± SEM (*n* = 6–8). *p < 0.05 vs. Ctrl.

The changes in the microbiome bacteria with proportions ≥9% at the six levels (phylum, class, order, family, genus, and species) are shown in Table 2 and Figure 2. 16S rRNA sequencing of fecal samples revealed that antibiotic intervention led to a significant reduction in microbial diversity by over 90% (Table 2). Phylum-level analysis showed a significant microbial compositional alteration in AD models, characterized by reduced *Bacteroidetes* and elevated *Firmicutes*, suggesting a potential shift in microbiota composition associated with AD pathogenesis. At the order level, we observed a similar change in the *Bacteroidales*/*Clostridiales* ratio (*p* < 0.01). In AD mice, *Bacteroidia* was significantly under-represented, and *Clostridia was* significantly over-represented at the class level. The abundance of *Porphyromonadaceae* did not change significantly in the AD group. In contrast, at the family, genus, and species levels, *Prevotellaceae, Rikenellaceae, Alistipes, Alloprevotella*, and *Alloprevotella rava* were significantly reduced, whereas *Lachnospiraceae*, and *Clostridium XlVa* were significantly over-represented. To further identify the specific commensal bacteria associated with AD, we compared the microbial compositions of the Ctrl and AD groups. LDA demonstrated significant over-representation of *Bacteroidetes, Bacteroidia, Bacteroidales, Alistipes, Rikenellaceae, Prevotellaceae, Comamonadaceae, Anaerotruncus, Alphaproteobacteria, Anaerovorax, Clostridiales, Butyrivibrio, Paraprevotella, Deferribacteres, Deferribacteraceae, Mucispirillum, Deferribacteres, and Deferribacterals* in the Ctrl group and significant over-representation of *Firmicutes, Clostridiales, Clostridia, Lachnospiraceae, Clostridium XlVa, Acetatifactro*, and *Sporobacter* in the AD group (Figure 3B). The LDA effect sizes (Figure 3C) were consistent with the microbiome composition analysis. The above data showed a significant change in the ratio of the *Bacteroides/Firmicutes* in AD mice.

**Table 2:** Relative abundance at phylum, order, class, family, genus, and species level in gut microbiota composition.

**Taxonomic rank**	**Bacterial**	**Ctrl (mean ±SD)**	**AD (mean ±SD)**	**ABX (mean ±SD)**	**AD + ABX (mean ±SD)**
Phylum	*Bacteroidetes*	69.43 ± 7.62	40.09 ± 4.39^**^	1.70 ± 0.93	1.65 ± 0.54##
	*Firmicutes*	26.74 ± 6.54	54.64 ± 5.30^**^	1.00 ± 0.77	1.70 ± 0.65##
Order	*Bacteroidales*	69.32 ± 6.80	40.51 ± 5.96^**^	1.30 ± 0.74	1.27 ± 0.67##
	*Clostridiales*	26.35 ± 5.60	52.34 ± 5.21^**^	1.19 ± 0.93	1.10 ± 0.94##
Class	*Bacteroidia*	69.32 ± 6.80	40.51 ± 5.96^**^	1.09 ± 0.64	1.27 ± 0.67##
	*Clostridia*	26.79 ± 5.94	52.35 ± 5.21^**^	1.24 ± 0.39	1.24 ± 0.93##
Family	*Lachnospiraceae*	19.30 ± 4.57	41.48 ± 5.13^**^	1.66 ± 0.79	1.78 ± 1.11##
	*Porphyromonadaceae*	37.16 ± 4.70	32.50 ± 5.14	2.28 ± 0.83	2.42 ± 0.91##
	*Prevotellaceae*	17.08 ± 3.90	2.99 ± 1.46^**^	1.18 ± 0.44	1.07 ± 0.47##
	*Rikenellaceae*	10.76 ± 2.00	1.62 ± 0.51^**^	0.87 ± 0.61	0.49 ± 0.23##
Genus	*Alistipes*	9.26 ± 2.25	0.68 ± 0.28^**^	0.06 ± 0.02	0.05 ± 0.02##
	*Alloprevotella*	11.38 ± 2.57	2.23 ± 0.80^**^	0.78 ± 0.29	0.54 ± 0.48##
	*Clostridium_XlVa*	6.71 ± 1.28	16.72 ± 5.31^**^	1.70 ± 0.65	1.77 ± 1.25##
	*Unclassified*	51.97 ± 4.68	58.25 ± 4.25^*^	6.79 ± 1.45	6.38 ± 1.34##
Species	*Alloprevotella_rava*	12.60 ± 2.36	1.98 ± 0.66^**^	0.41 ± 0.21	0.43 ± 0.35##
	*Unclassified*	80.44 ± 3.36	83.62 ± 1.03^*^	5.79 ± 1.43	6.57 ± 1.60##

The table only lists the key bacterial that account for ≥ 9% and have statistical differences (*P* < 0.05) compared to the Ctrl group (^*^) or AD group (#).^*^P < 0.05, ^**^P < 0.01 vs. Ctrl; #*P* < 0.05, ##*P* < 0.01 vs. AD.

### 3.3 Differential metabolite screening between Ctrl and AD mice

Partial least squares discriminant analysis (PLS-DA) and PCA model of Ctrl and AD samples showed good stability and repeatability (Figures 3A, B). Differential metabolite screening revealed significant alterations between two groups, as shown in Figure 3C and Table 3. Specifically, five metabolites were markedly upregulated, while six were notably downregulated. Compared with the Ctrl group, the expression levels of 3,4-Dihydroxymandelic acid, 3-Mercaptolactic acid, Indole-3-propionic acid, Pentacosanoic acid, Amygdalin, and Acetate were significantly under-represented in AD mice, while 12-Ketolithocholic acid acetate, Etiadienic Acid, Stearoylcarnitine, Palmitoylcarnitine, and Arachidonic acid were significantly over-represented. Among the differential metabolite, the expression of acetate was the highest. We further examined the SCFA levels in AD mice using LC-MS analysis. Gut microbiota-derived SCFA profiles revealed a marked decrease in acetate concentrations along with total SCFA levels in AD model mice, whereas propionate, butyrate, valerate, caproate, isobutyrate, and isovalerate remained at comparable levels relative to healthy controls (Figure 3D). These observations imply that acetate, serving as a principal microbial metabolic product, may function as a critical modulator in the pathophysiological progression of AD.

**Table 3:** Differential metabolite screening results.

**Metabolite**	**Class**	**Ctrl (mean ±SD)**	**AD (mean ±SD)**	**Fold change**	***p-value***
12-Ketolithocholic acid acetate, Methyl Ester	Bile acids	0.107 ± 0.020	0.142 ± 0.031	1.329	0.044
Etiadienic acid	Bile acids	0.002 ± 0.0002	0.003 ± 0.001	1.282	0.015
Stearoylcarnitine (C18)	Carnitines or Acyl carnitines	0.0002 ± 0.0001	0.0006 ± 0.0007	3.822	0.039
Palmitoylcarnitine (C16)	Carnitines or Acyl carnitines	0.006 ± 0.002	0.048 ± 0.046	7.438	0.002
Arachidonic acid	Fatty acids	0.046 ± 0.028	0.200 ± 0.108	4.082	0.008
3,4-Dihydroxymandelic acid	Benzenoids	0.050 ± 0.012	0.036 ± 0.007	0.717	0.023
3-Mercaptolactic acid	Carbohydrates	4.501 ± 0.814	3.340 ± 0.689	0.742	0.019
Indole-3-propionic acid	Indoles and derivatives	0.011 ± 0.003	0.006 ± 0.003	0.588	0.031
Pentacosanoic acid	Fatty acyls	1.789 ± 0.277	1.452 ± 0.147	0.811	0.023
Acetate	Fatty acids	8.573 ± 1.505	6.434 ± 0.832	0.750	0.012
Amygdalin	Carbohydrates	0.004 ± 0.0004	0.003 ± 0.0003	0.821	0.005

### 3.4 Acetate supplementation protects against AD-like skin lesions

**Figure 4:**
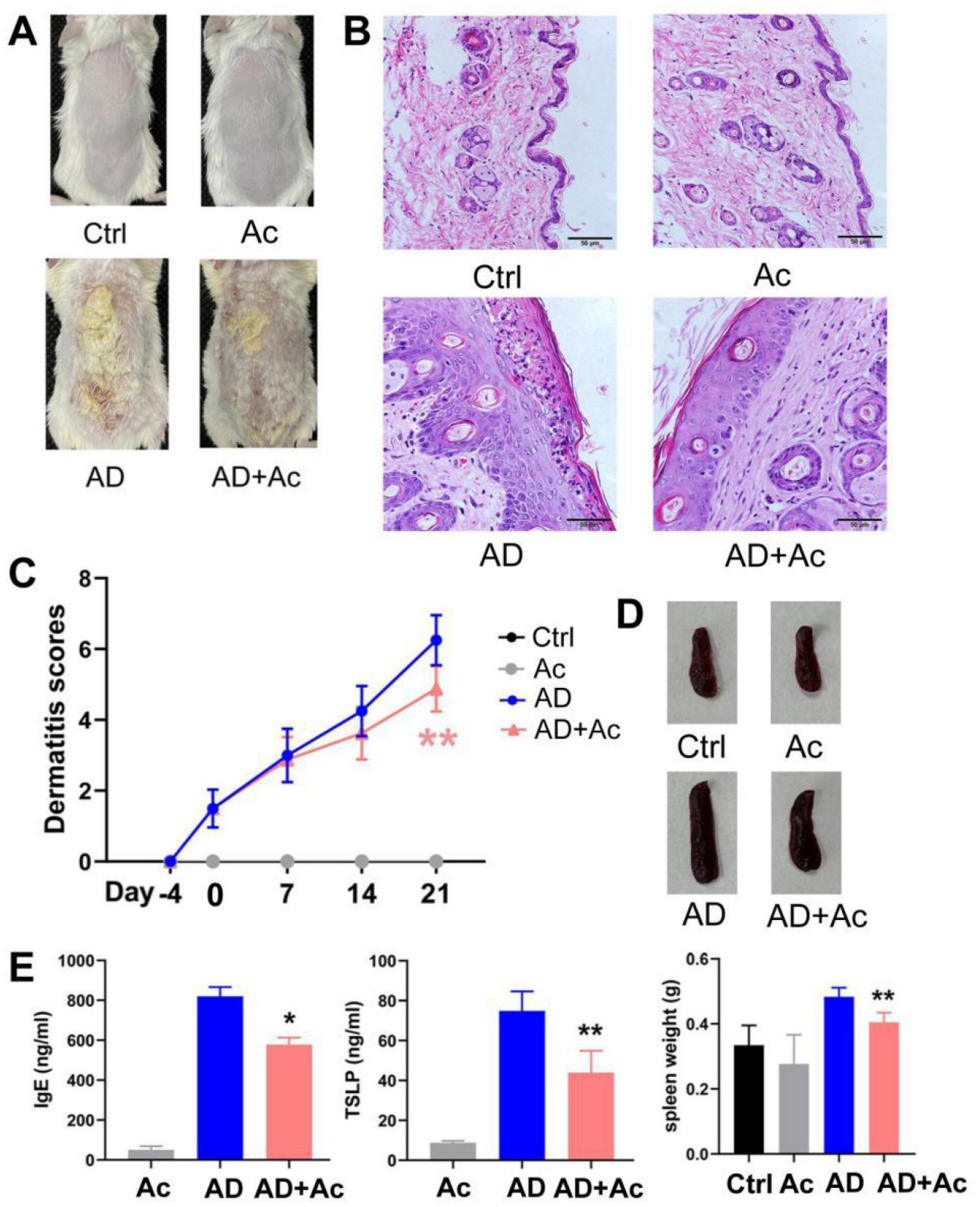
Acetate administration significantly reduced DNCB-induced AD-like skin damage. **(A)** Photograph of the dorsal skin of mice. **(B)** H&E staining for histopathological features (bar length = 50 μm). **(C)** Dermatitis scores of the mice after acetate treatment (200 mM for 4 weeks). **(D)** The spleen pictures and weight of mice. **(E)** Levels of IgE and TSLP in the peripheral blood of mice after acetate treatment. The results are presented as mean ± SEM (*n* = 6–8). Control group (Ctrl), AD model group (AD), Acetate group (Ac), AD+Acetate group (AD+Ac), *p < 0.05, **p < 0.01 vs. AD.

We explored whether acetate is involved in the mechanism of AD. Currently, no animal model for AD fully replicates the complexity of human clinical conditions. However, DNCB-induced AD mice are widely used due to their skin lesion characteristics and Th2 polarization, which closely resemble those of human AD, making them suitable for the objectives of this study. After supplementing with acetate, the acetate content in the Ac group significantly increased, and no significant difference was observed between the AD+Ac and Ctrl group, indicating that the supplementation was effective (data not shown). Additionally, acetate supplementation did not cause any sensitivity reaction or redness in the skin of mice (Figures 4A, B).

**Figure 5:**
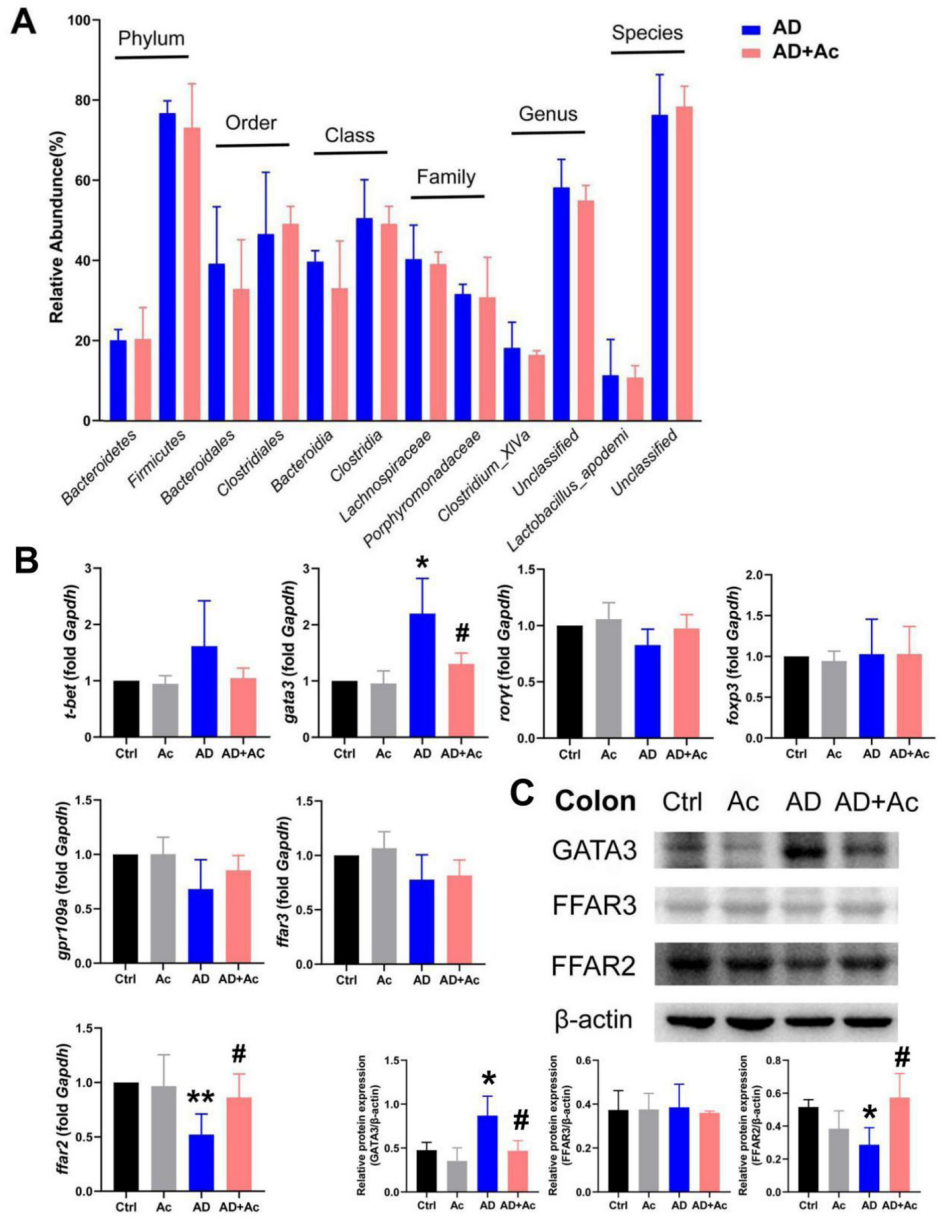
FFAR2 is essential for acetate administration in DNCB-induced AD. **(A)** Comparison of six levels proportional abundance of feces from mice under AD and acetate pre-treatment (AD+Ac) group (relative abundance). **(B)** mRNA expression of t-bet, gata3, rorγt, foxp3, ffar2, ffar3, and gpr109a in the colon of the indicated mice. **(C)** Relative Gata3, FFAR3, and FFAR2 protein expression in colon tissues of mice. The results are presented as mean ± SEM (*n* = 6–8). ^*^p < 0.05, ^**^p < 0.01 vs. Ctrl; #*p* < 0.01 vs. AD.

Acetate supplementation alleviated the inflammation symptoms of AD mice, as evidenced by thinner skin, decreased lesion, and significantly reduced spleen weight (Figures 4A, B, D). As shown in Figure 4B, the AD group exhibited significant epidermal pathology compared to controls, including increased epidermal thickness, spinous layer hyperplasia, spongiotic edema with inflammatory infiltration, and impaired keratinization. In the AD+Ac group, these pathological changes were measurably ameliorated, as evidenced by reduced epidermal hyperplasia, partially restored keratinization, attenuated spongiosis, and diminished inflammatory cell infiltration. At the end of the intervention period, the AD+Ac mice exhibited significantly lower dermatitis scores than the AD group did (Figure 4C). Acetate supplementation in the AD group significantly decreased the IgE and TSLP levels (Figure 4E). Thus, acetate might be a critical modulator in DNCB-induced AD mice. To better understand the interaction between gut microbiota and acetate, we conducted 16S rRNA sequencing analysis to characterize microbial community alterations following acetate supplementation. We found that acetate supplementation did not affect gut microbiota composition or relative abundances (Figure 5A). The gut microbiota can affect the level of acetate, but an increase in acetate concentration within a certain concentration range does not affect the gut microbiota, indicating that the gut microbiota and acetate may have a unidirectional causal relationship.

### 3.5 FFAR2 is essential for acetate supplementation in DNCB-induced AD

SCFAs have been reported to activate FFAR3 (previously termed GPR41), FFAR2 (previously termed GPR43), and GPR109a, with FFAR2 agonists shown to ameliorate DNCB-induced AD in mice (Moniri and Farah, 2021; Kang and Im, 2020). We examined the relative expression of SCFAs receptors (*ffar2, ffar3, gpr109a*) and T-helper cell transcription factors *t-bet* (Th1, T helper type 1), *gata3* (Th2), *ror*γ*t* (Th17), and *foxp3* (T regulatory) following acetate supplementation. The results revealed that *ffar2* mRNA expression was reduced in the colon of AD mice (*p* < 0.01), whereas *gata3* mRNA expression was significantly increased (Figure 5B). After acetate supplementation, *ffar2* mRNA expression was significantly upregulated, and *gata3* mRNA expression was significantly downregulated. No significant differences were detected in the mRNA expression of *ffar3, gpr109a, t-bet, ror*γ*t*, and *foxp3* in the colon across all experimental groups. At the protein level, GATA3 expression was markedly upregulated in AD mice relative to controls, whereas FFAR2 levels were significantly diminished, and FFAR3 expression remained unaltered (Figure 5C). Hence, the therapeutic mechanism of acetate in AD is related to the FFAR2 and Th2 (Gata3) pathways.

## 4 Discussion

Several studies have revealed that gut microbiota-derived metabolites can enter the circulation and produce systemic effects, including on the skin. Despite this, the underlying mechanisms and relationship linking microbiota and SCFAs to the pathogenesis of AD remain poorly understood, highlighting the need for further investigation (Moniaga et al., 2022; Moniri and Farah, 2021). Here, we constructed a gut microbiota depletion mouse model and found that disrupting gut microbiota exacerbated symptoms in AD mice. Furthermore, we described the changes in the abundance of gut microbiota and metabolic profiles in Ctrl and AD murine models. These data can serve as a foundation for in-depth studies on microbial communities and metabolic levels, and inform biomedical research projects focused on the development of probiotics, functional foods, diagnostics, and drugs. 16S rRNA analysis revealed significant alterations in the *Bacteroides/Firmicutes* ratio in AD mice, providing a foundation for further investigation of gut microbiota in AD mouse models. At different levels, *Bacteroidales* and *Bacteroidia* were significantly downregulated, while *Clostridiales* and *Clostridia* were significantly upregulated in the AD group, which is consistent with the change in the *Bacteroides/Firmicutes* ratio at the phylum level. We also noticed significant changes in the *Alloprevotella rava* and *Alistipes* abundance that correlated with depressive states in the mice (Stojanov et al., 2020). Patients with AD frequently experience depression due to poor skin conditions and impaired quality of life. Anxiety and depression are closely linked to gut bacterial dysbiosis, which correlated with an increased *Bacteroidetes*/*Firmicutes* ratio (Luqman et al., 2024).

Research has confirmed that patients with AD have low fecal SCFA concentrations (Reddel et al., 2019; Wang et al., 2022). In this investigation, we characterized different metabolites using HM700 high-throughput metabolomics, and found that total SCFA and acetate levels were significantly decreased in the AD group. We also predicted that acetate might be an important metabolic marker for AD. Previous literature revealed that *Bacteroidetes* of the phylum *Bacteroides* were capable of producing high levels of acetate and propionate, while *Firmicutes* produced high levels of butyrate (Macfarlane and Macfarlane, 2003). Therefore, the decrease of *Bacteroides* can significantly reduce acetate concentrations, consistent with the results of 16S rRNA sequencing. We observed a decreasing trend of butyrate in AD mice, but the difference was not significant. Moreover, we conducted a butyrate supplementation experiment and did not observe any improvement in mouse skin lesions (data not shown). These results connect SCFAs, such as acetate, with high-abundance gut bacteria, particularly *Bacteroides*, suggesting the potential therapeutic effects of acetic acid in AD by restoring gut microbiota and maintaining gut health. Moreover, our results demonstrated that acetate supplementation alleviates DNCB-induced AD-like skin damage, which is related to the presence of the FFAR2 and the Th2 (Gata3) pathways, but not to FFAR3 and GRP109a. Previous studies have shown that SCFAs interact with both shared and specific cellular receptors; acetate predominantly activates FFAR2, whereas butyrate exhibits greater selectivity for GPR109a (Niu et al., 2023). AD is widely recognized as an inflammatory disorder driven primarily by Th2 cell-mediated immune responses (Adhikary et al., 2021). Despite extensive research on Th2 cell involvement in AD, few studies have reported the specific role of acetate in modulating Th2 cell-associated immune inflammatory response. This gap warrants further investigation into microbial metabolite-Th2 cell crosstalk.

In this study, we cannot rule out the possibility that acetate supplementation also activates other tissues, such as intestinal epithelial cells, thereby influencing FFAR2 expression, which may indirectly affect the production of Gata3, IgE, and TSLP. AD is a chronic, recurrent skin disorder that often necessitates long-term pharmacological treatment, raising concerns among patients regarding safety and potential side effects. Acetate, a key microbial metabolite produced by the human gut microbiota, has a wide range of applications in the field of food. High-fiber diets significantly increase acetate levels (Marques et al., 2017). A cross-sequential study showed high fiber intake (~98.25 g/serving/week) significantly lowered the associated risks for AD and house dust mite allergy (Lim et al., 2025). Consequently, future preventive strategies for clinical AD could potentially involve dietary interventions, such as acetate-related supplements or increased intake of high-fiber foods. Further elucidation of the interactions among gut microbiota, acetate, and Th2-associated pathways in AD could support the development of dietary supplements for the prevention or treatment of this disease.

## 5 Conclusions

**Figure 6:**
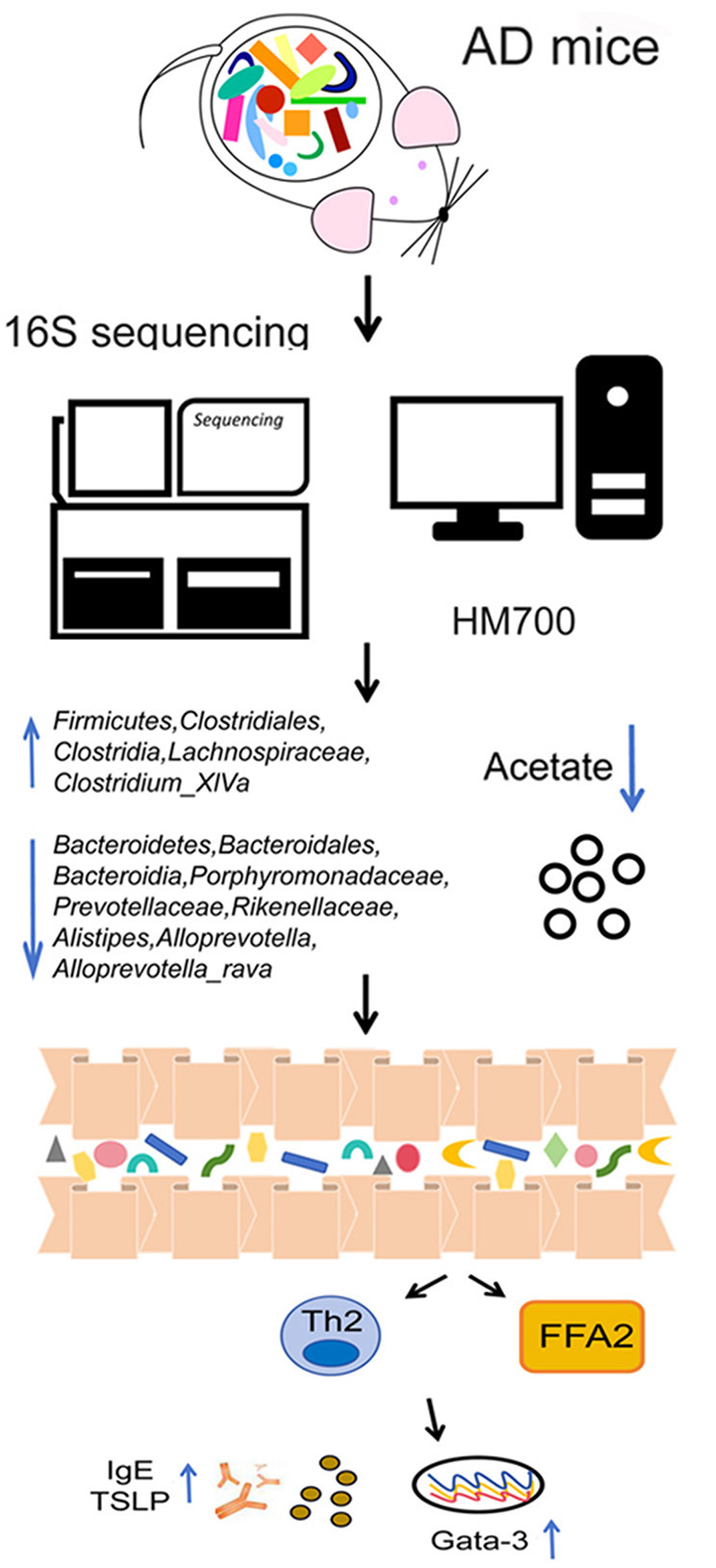
Schematic diagram illustrating a potential mechanism for the protective role of acetate on DNCB-induced AD. The Bacteroidetes/Firmicutes ratio and low expression of acetate play important regulatory roles in AD. Acetate can alleviate AD by binding to the receptor FFAR2 and inhibiting Th2-related factors.

Our data indicate that the *Bacteroidetes*/*Firmicutes* and low expression of acetate play important regulatory roles in AD. Acetate can alleviate DNCB-induced AD by binding to the receptor FFAR2 and inhibiting Th2-related factors (Figure 6). This mechanism may be relevant for treating and preventing AD and other skin inflammatory diseases.

## Data Availability

The original contributions presented in the study are publicly available. This data can be found in here: http://www.ncbi.nlm.nih.gov/bioproject/1295353 (16s rRNA gene sequencing), 10.6084/m9.figshare.29626133 (Metabonomic data).
