# Supplementary figures and images for: Gut microbiota metabolite acetate mediates free fatty acid receptor 2 expression to alleviate atopic dermatitis
Source: Front Microbiol. 2025 Aug 12;16:1595532. doi: 10.3389/fmicb.2025.1595532 (PMC12379089; doi:10.3389/fmicb.2025.1595532)

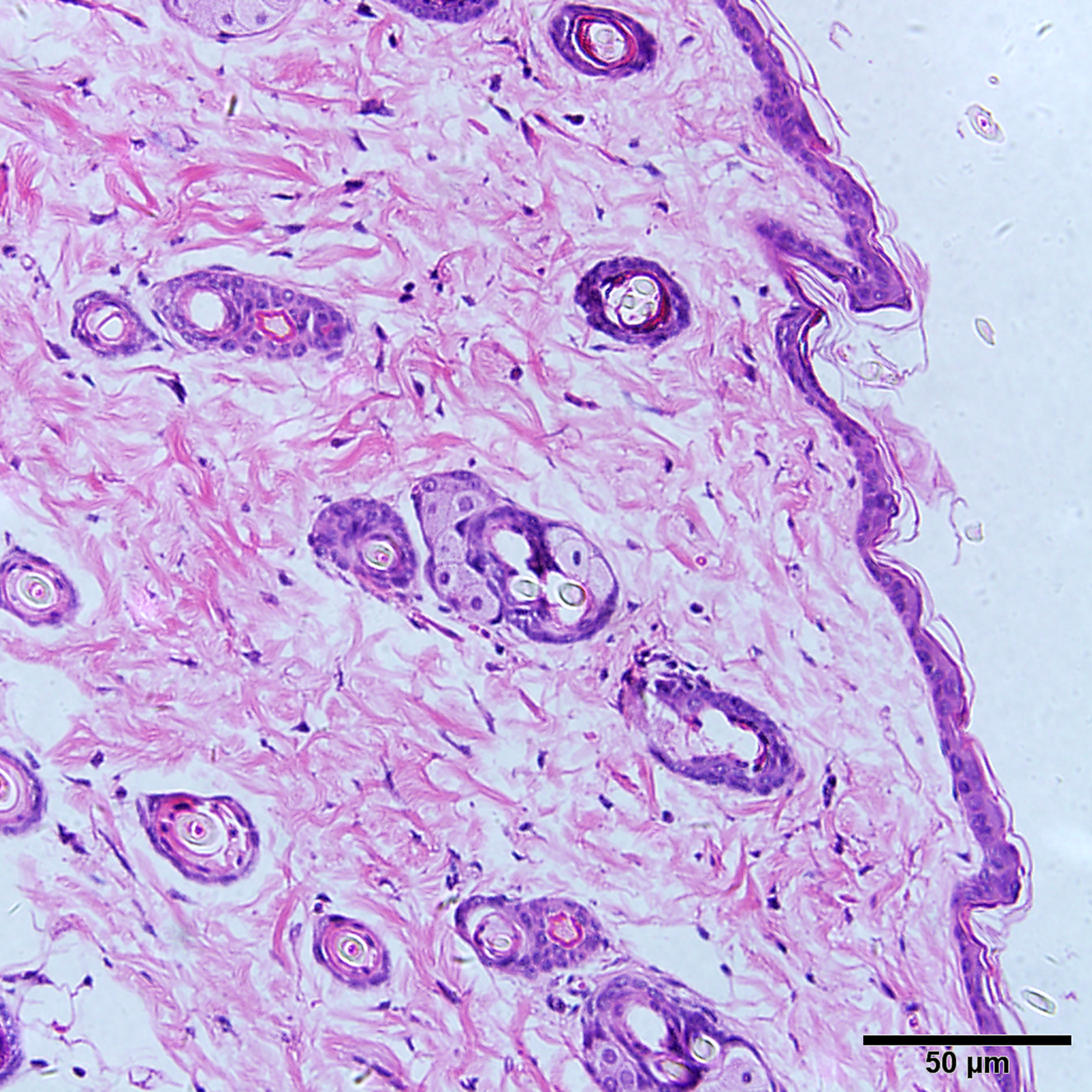

Supplement: Supplementary file 1 [file Data_Sheet_1.zip › H&E staining for histopathologicafeatures/AC.tif]

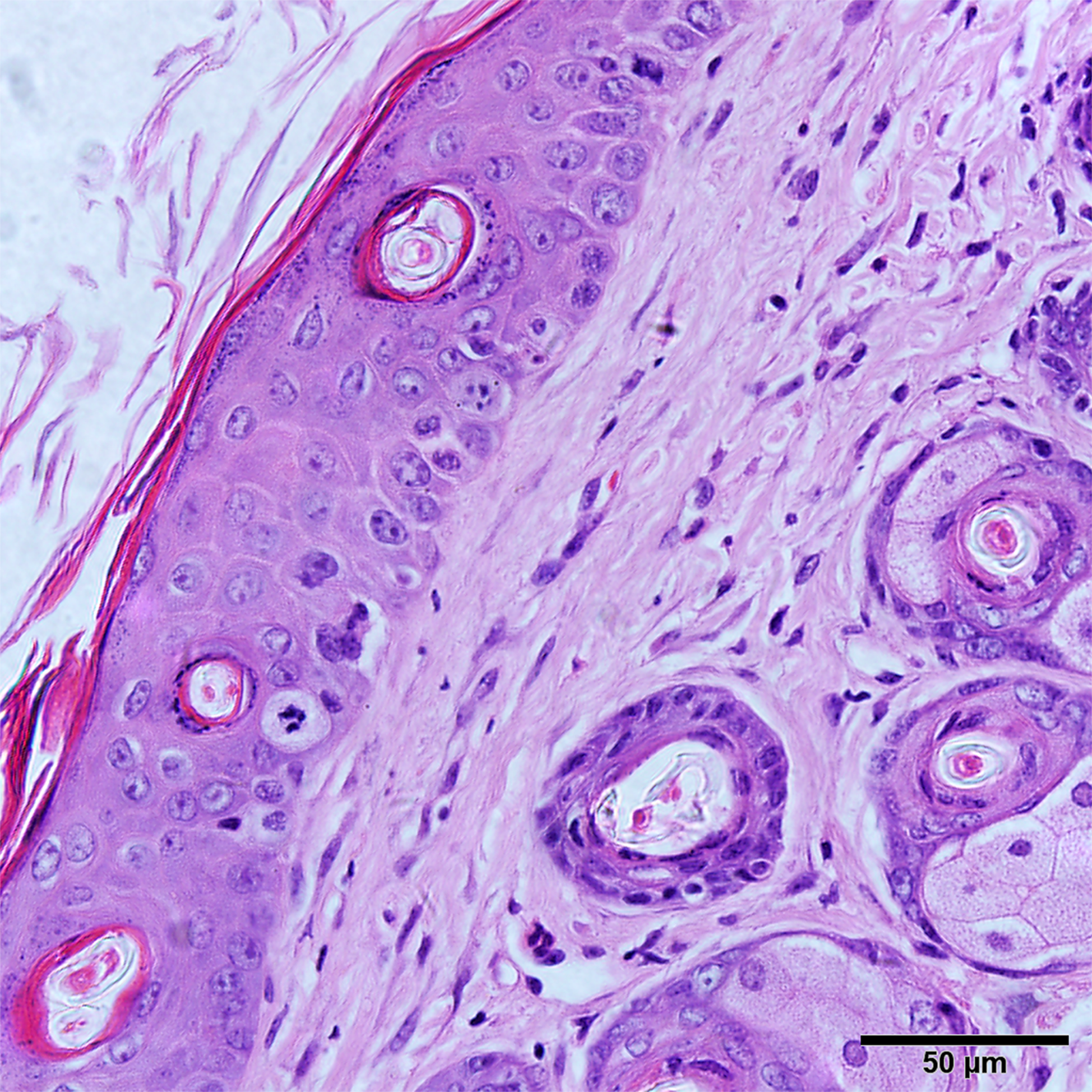

Supplement: Supplementary file 1 [file Data_Sheet_1.zip › H&E staining for histopathologicafeatures/AD+Ac.tif]

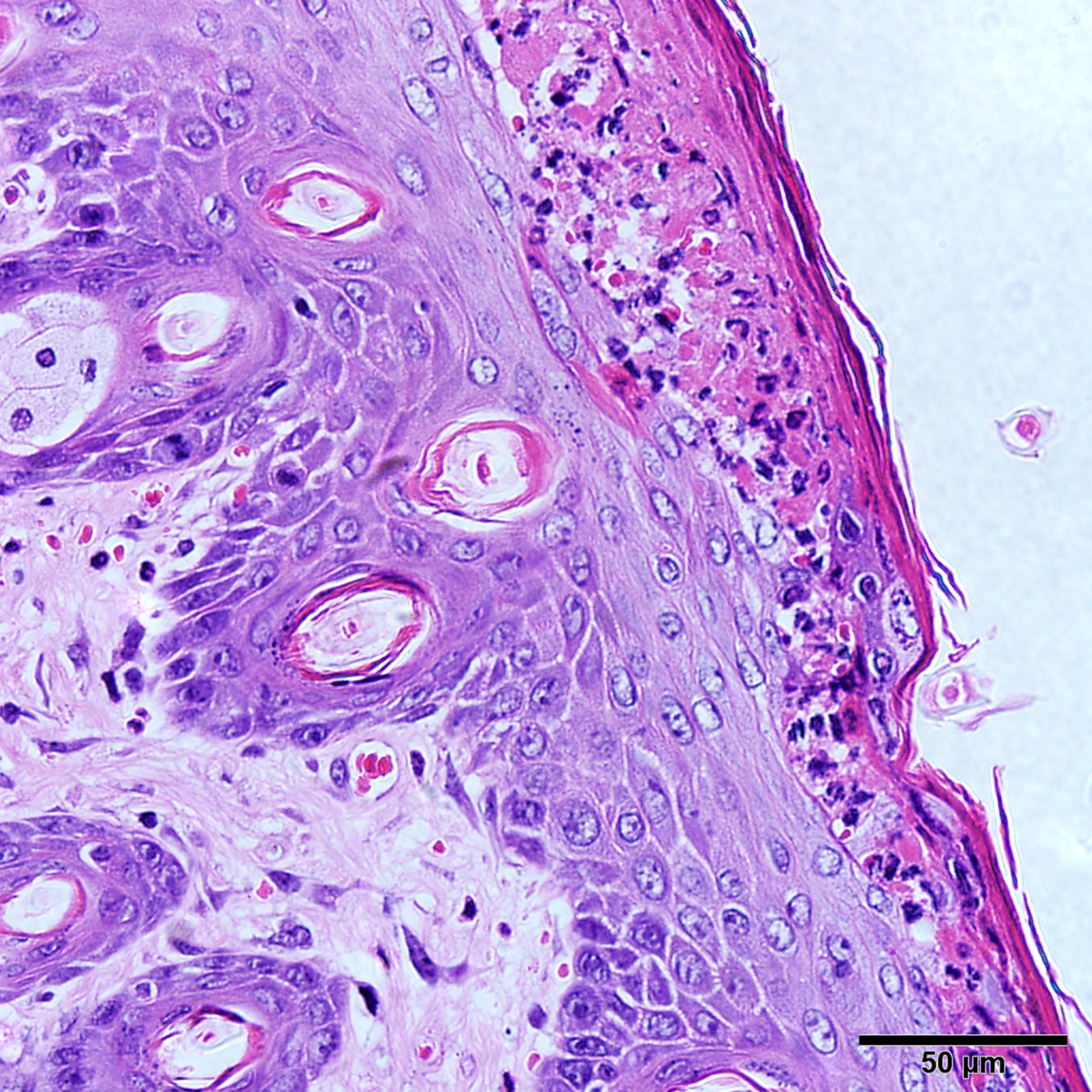

Supplement: Supplementary file 1 [file Data_Sheet_1.zip › H&E staining for histopathologicafeatures/AD.tif]

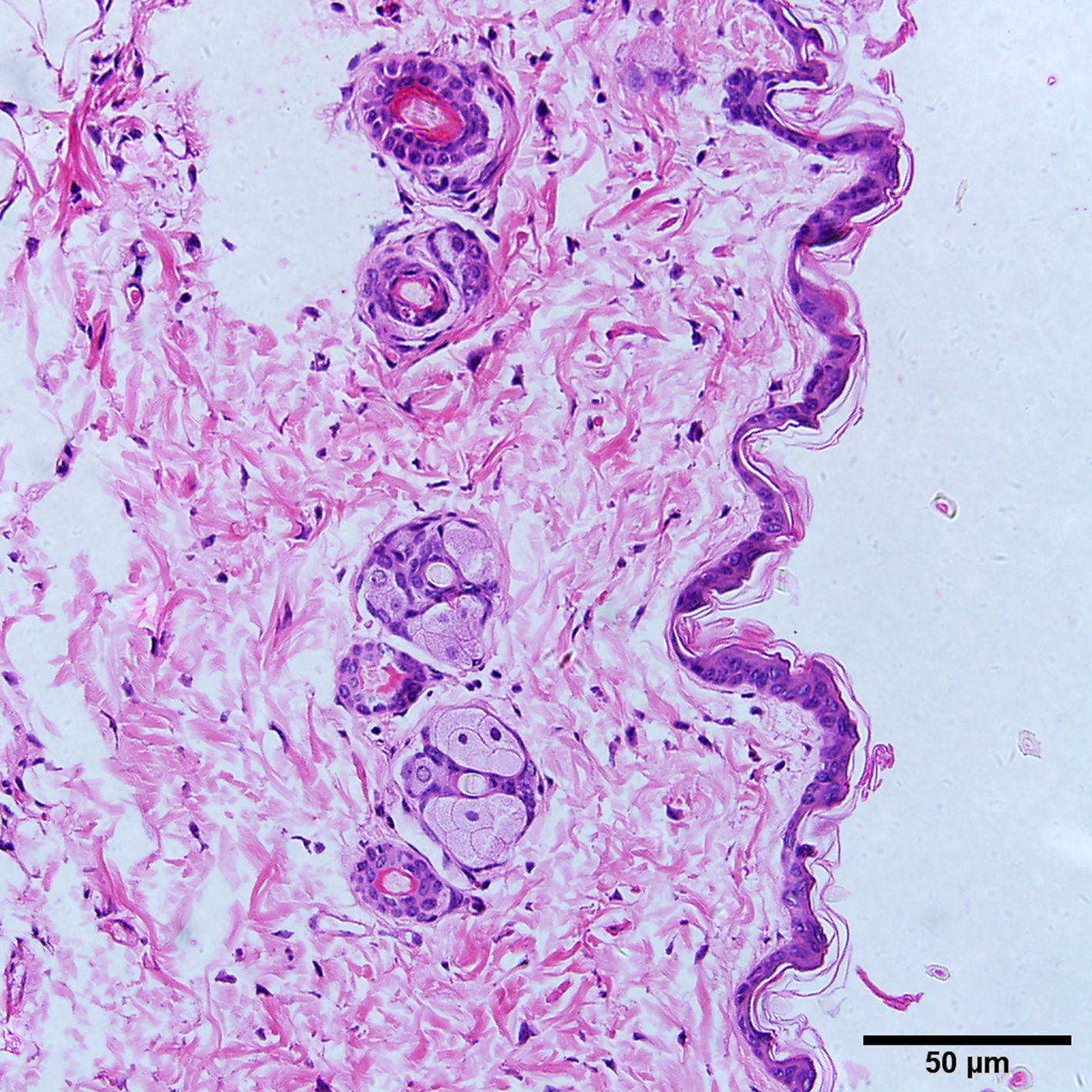

Supplement: Supplementary file 1 [file Data_Sheet_1.zip › H&E staining for histopathologicafeatures/Ctrl.tif]

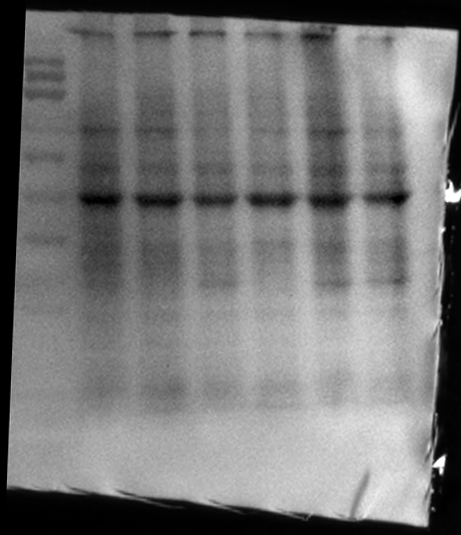

Supplement: Supplementary file 2 [file Data_Sheet_2.zip › WB Raw Data/FFAR2.tif]

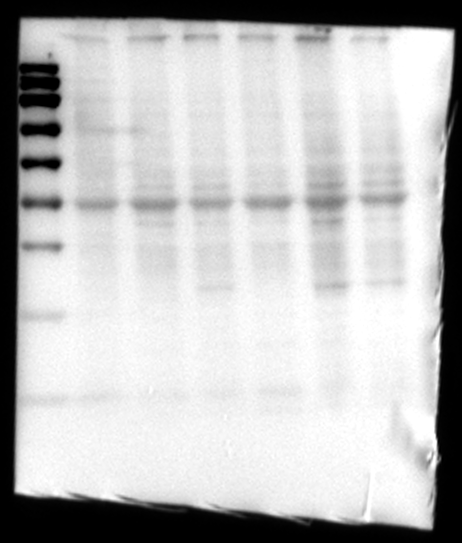

Supplement: Supplementary file 2 [file Data_Sheet_2.zip › WB Raw Data/FFAR3.tif]

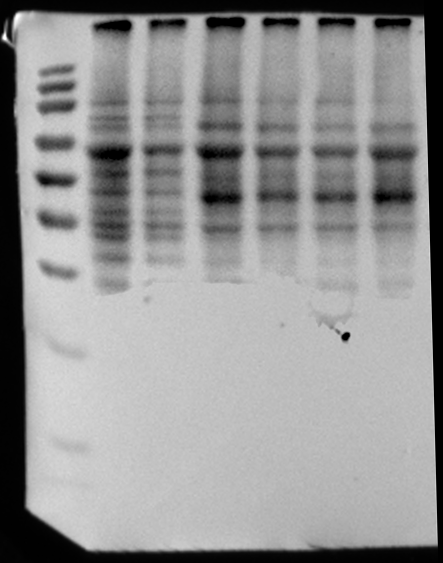

Supplement: Supplementary file 2 [file Data_Sheet_2.zip › WB Raw Data/GATA-3.tif]

Figure 5C

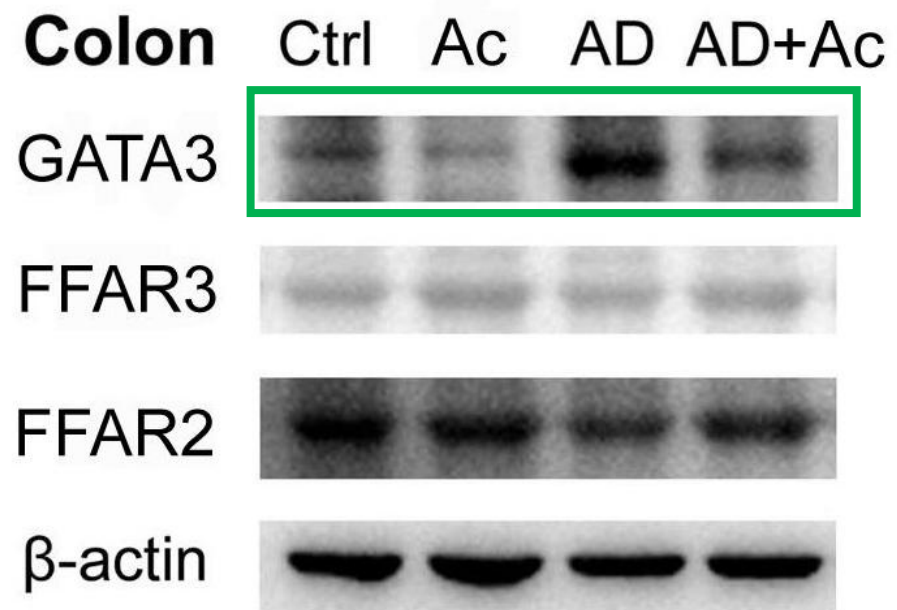

Raw data

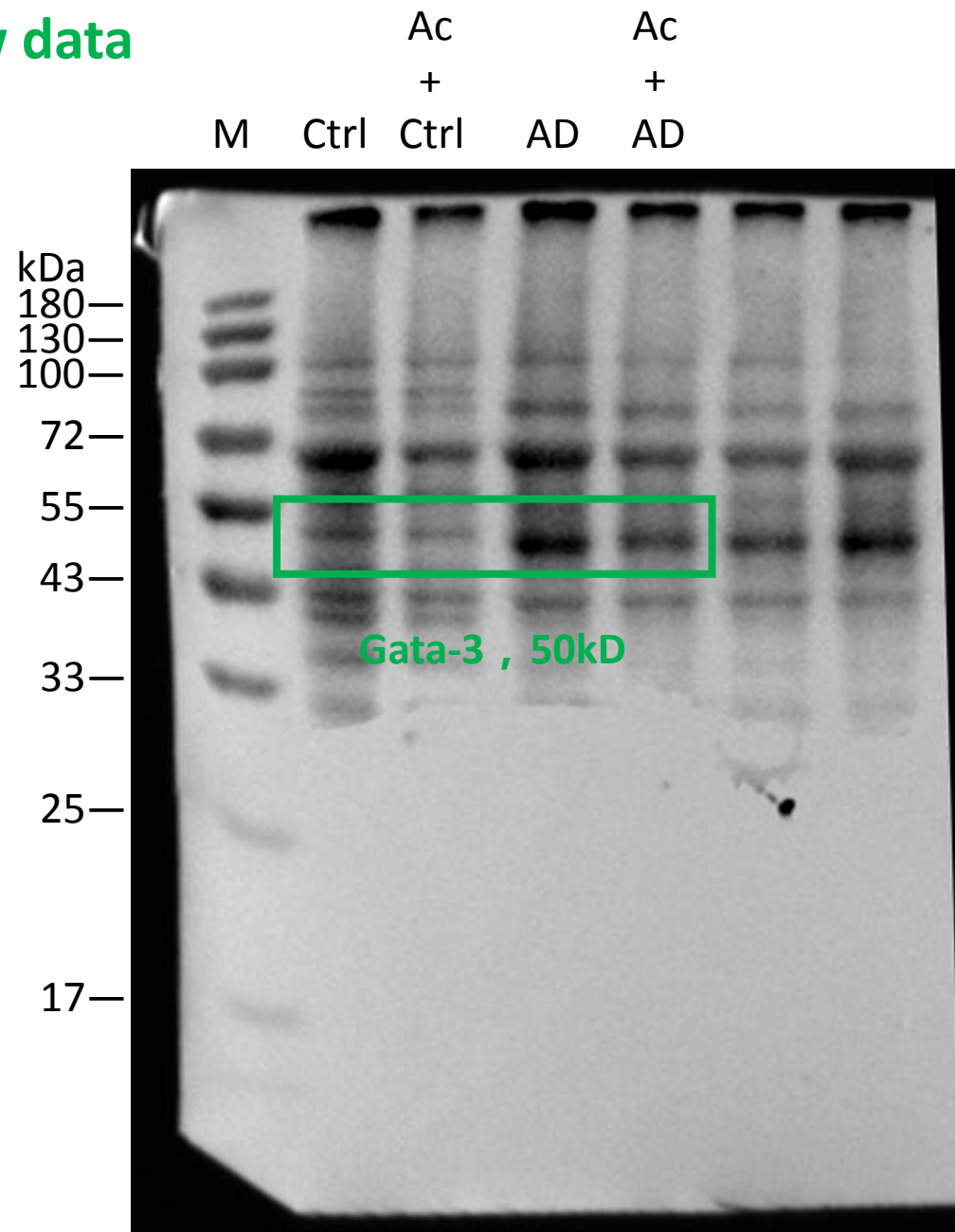

Figure 5C

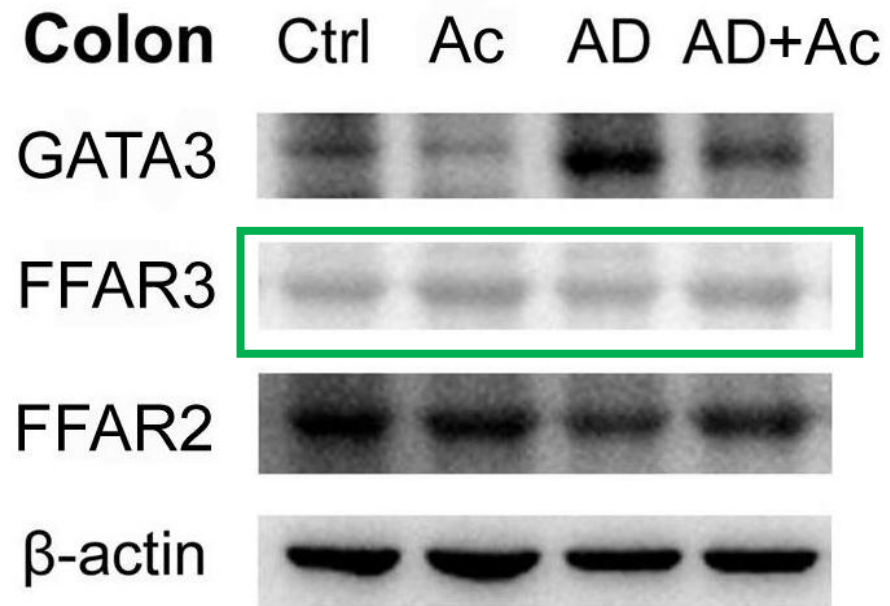

Raw data

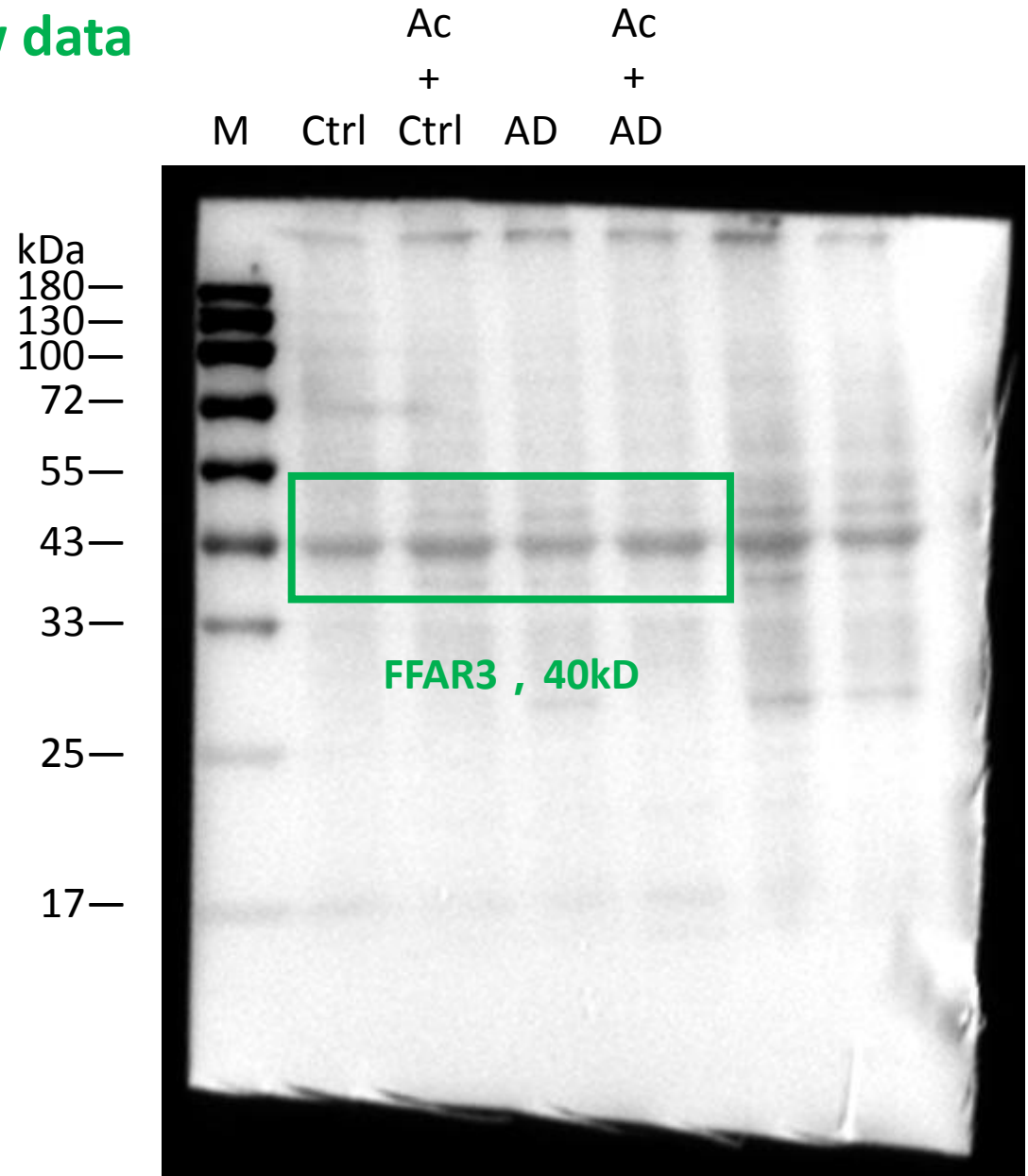

Figure 5C

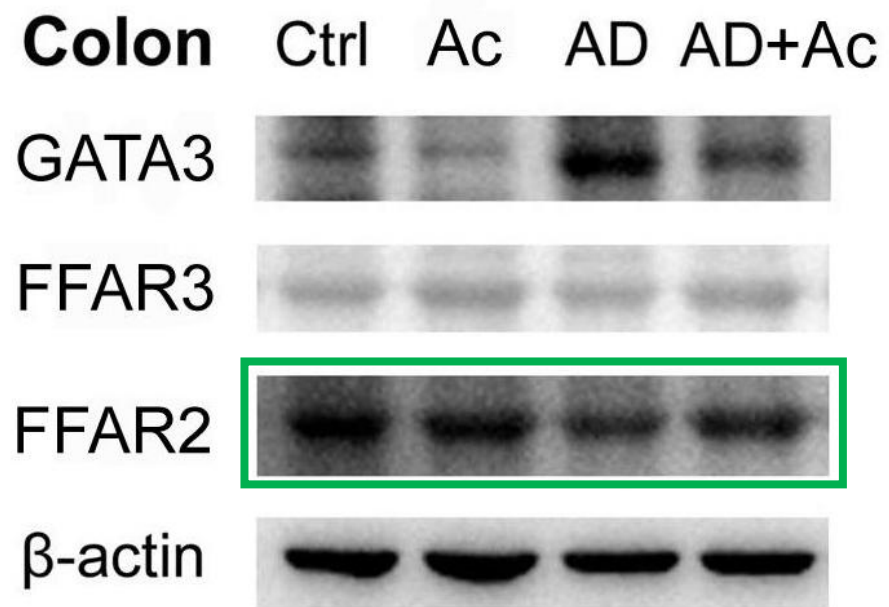

Raw data

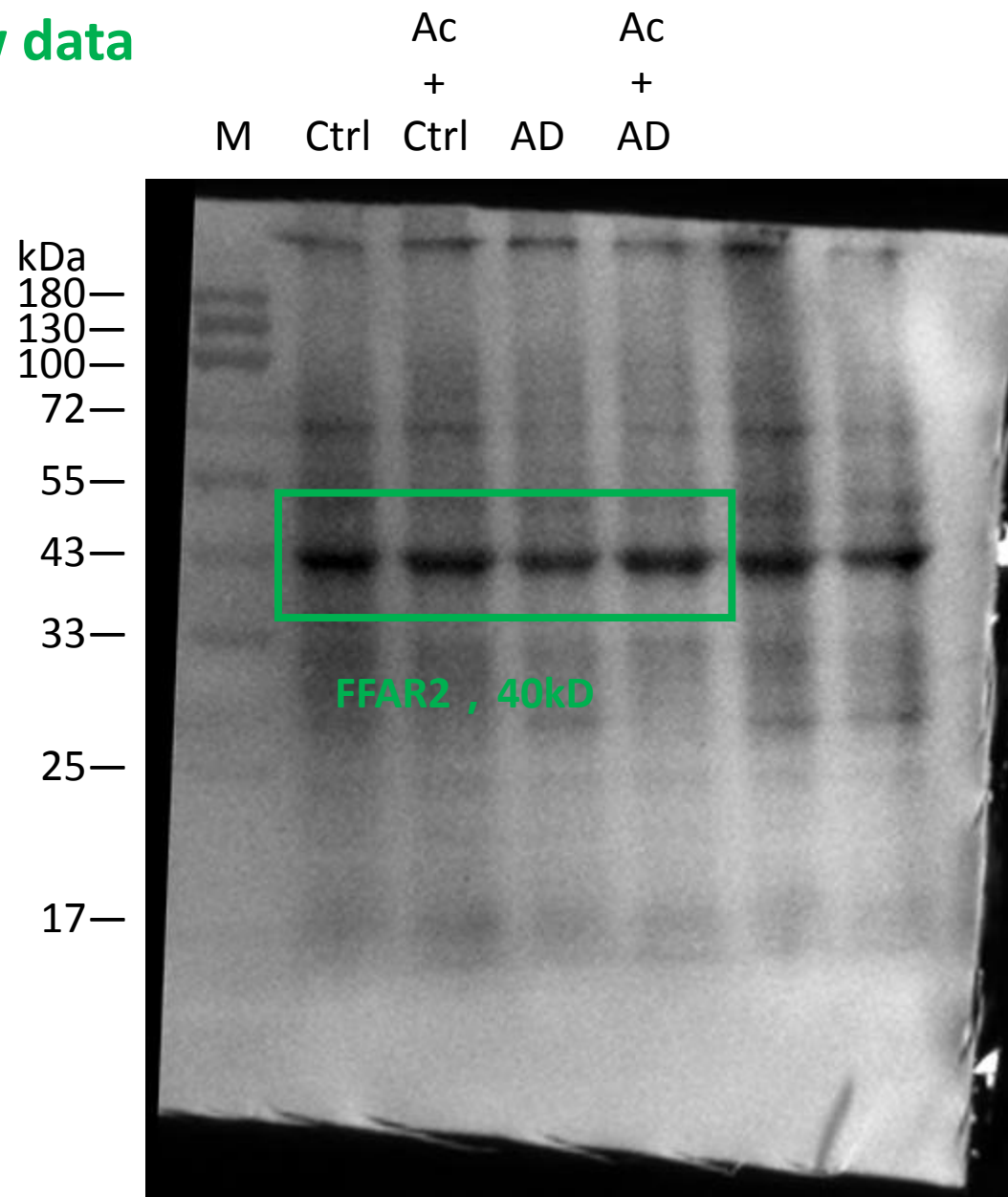

Figure 5C

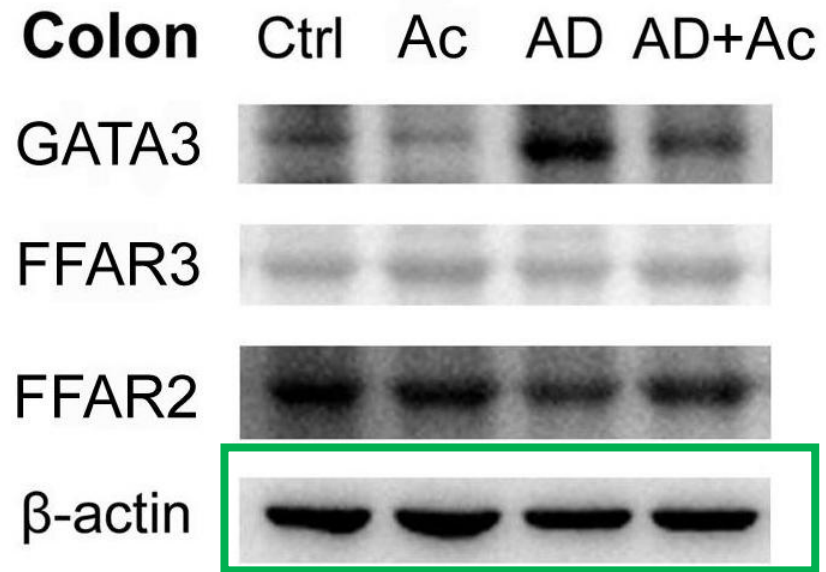

Raw data

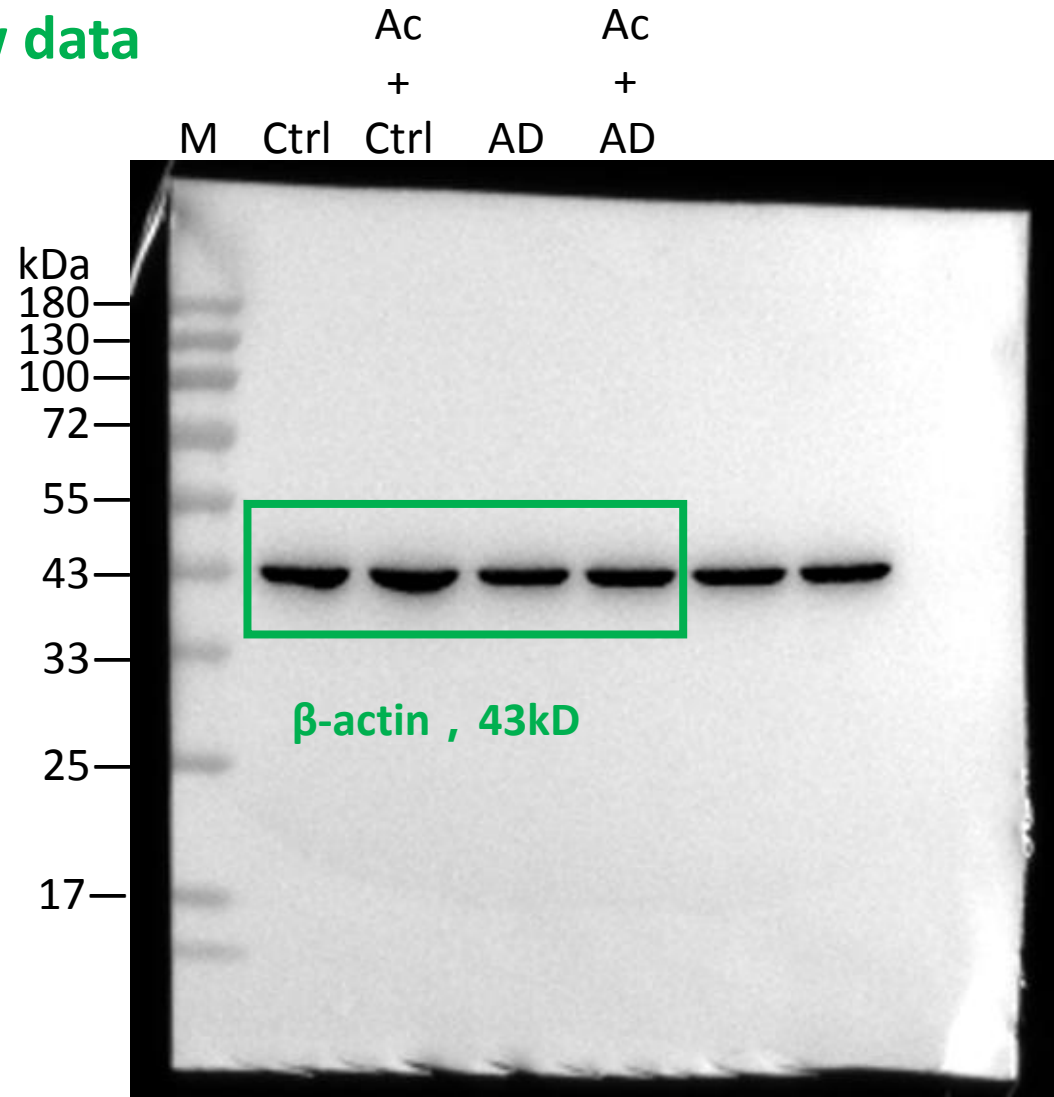

Supplement: Supplementary file 2 [file Data_Sheet_2.zip › WB Raw Data/WB Raw Data.pdf]

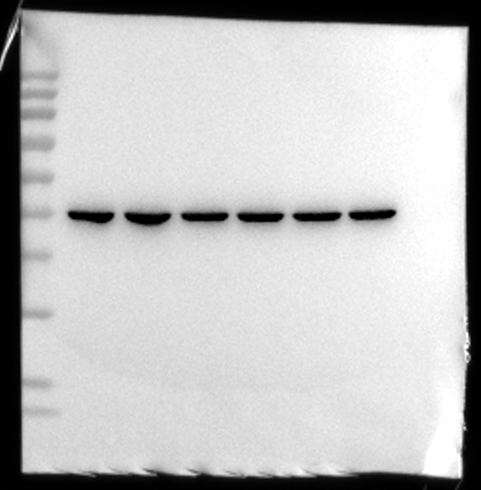

Supplement: Supplementary file 2 [file Data_Sheet_2.zip › WB Raw Data/β-actin.tif]
